# What is new in microcirculation and tissue oxygenation monitoring?

**DOI:** 10.1007/s10877-022-00837-x

**Published:** 2022-03-11

**Authors:** Ilonka N. de Keijzer, Dario Massari, Marko Sahinovic, Moritz Flick, Jaap Jan Vos, Thomas W. L. Scheeren

**Affiliations:** 1 University Medical Center Groningen, Groningen, The Netherlands; 2 Department of Anesthesiology, Center of Anesthesiology and Intensive Care, University Medical Center Hamburg-Eppendorf, Hamburg, Germany

**Keywords:** Microcirculation, Tissue oxygenation, Near-infrared spectroscopy, Monitoring, Tissue perfusion, Plethysmography

## Abstract

Ensuring and maintaining adequate tissue oxygenation at the microcirculatory level might be considered the holy grail of optimal hemodynamic patient management. However, in clinical practice we usually focus on macro-hemodynamic variables such as blood pressure, heart rate, and sometimes cardiac output. Other macro-hemodynamic variables like pulse pressure or stroke volume variation are additionally used as markers of fluid responsiveness. In recent years, an increasing number of technological devices assessing tissue oxygenation or microcirculatory blood flow have been developed and validated, and some of them have already been incorporated into clinical practice. In this review, we will summarize recent research findings on this topic as published in the last 2 years in the *Journal of Clinical Monitoring and Computing *(*JCMC*)*.* While some techniques are already currently used as routine monitoring (e.g. cerebral oxygenation using near-infrared spectroscopy (NIRS)), others still have to find their way into clinical practice. Therefore, further research is needed, particularly regarding outcome measures and cost-effectiveness, since introducing new technology is always expensive and should be balanced by downstream savings. The JCMC is glad to provide a platform for such research.

## Introduction

Ensuring and maintaining adequate tissue oxygenation at the microcirculatory level might be considered the holy grail of optimal hemodynamic patient management. However, in clinical practice we usually focus on macro-hemodynamic variables such as blood pressure, heart rate, and sometimes cardiac output. Other macro-hemodynamic variables like pulse pressure or stroke volume variation are used as markers of fluid responsiveness. In recent years, an increasing number of technological devices assessing tissue oxygenation or microcirculatory blood flow have been developed and validated, and some of them have already been incorporated into clinical practice. In this review, we will summarize recent research findings on this topic as published in the last 2 years in the *Journal of Clinical Monitoring and Computing *(*JCMC*)*.*

## Microcirculation

Capillary refill time (CRT) is a simple and quickly measurable clinical sign to assess tissue perfusion. CRT is usually assessed visually and is thus highly prone to observer bias [1]. In a prospective observational study with 30 healthy volunteers and 19 patients in the emergency department, Shinozaki et al. [2] measured CRT using a modified pulse oximeter that applies a pressure of 400 mmHg on the fingertip for 5 s and transmits infrared light to detect changes in blood volume (defined as the capillary refill index), and compared it with CRT assessed by objective video analysis. The measurements in 30 healthy volunteers were performed at room temperature, after cooling the fingers to 15 °C, and after warming the fingertips to at least 30 °C. Bland–Altman analysis showed a mean of the differences (95% limits of agreement) of 1.01 (− 0.92 to 2.94) s for within-subject averages between automated capillary refill index and CRT. For individual measurements at room temperature the mean of the differences was 0.98 (− 0.72 to 2.67) s, with cooled fingertips 0.91 (− 3.46 to 5.28) s, and with rewarmed fingertips 1.15 (− 0.49 to 2.79) s. For the measurements in 19 patients in the emergency department the correlation coefficient with CRT was 0.76, which was lower than the reported correlation coefficient of 0.89 for the averaged measurements in healthy volunteers. For patients in the emergency department no Bland–Altman analysis was reported. Overall, the results indicate a systematic difference between the novel capillary refill index and CRT. These findings underline how challenging it is to obtain objective, reliable measurements of CRT. Whether this difference is clinically important—and whether using transmitted light for automated measurement of the capillary refill index is beneficial compared to conventional CRT remains unknown. In clinical practice, assessment of CRT by simply pressing on the fingertip is quick and easy to interpret and, therefore, unlikely being replaced by measurements that require additional technical equipment. Nonetheless, objective measurements of CRT—or the capillary refill index—are clinically important to increase comparability, especially in research. It will be interesting to see which role the capillary refill index will play in the future.

Laser speckle contrast imaging is a method to visualize and measure tissue perfusion from the speckle pattern that has been formed after it has been illuminated by coherent laser light [3]. We highlight two studies focusing on the use of laser speckle contrast imaging in patients having carotid endarterectomy [4, 5]. The first study by Motoyama et al. [4] investigated ocular blood flow using laser speckle contrast imaging in 19 patients having carotid endarterectomy under general anesthesia to measure ocular blood flow. Since the ophthalmic artery is a first main branch of the internal carotid artery it may reflect blood flow in the brain. Additionally, cerebral oxygen saturation was measured using near-infrared spectroscopy (NIRS) and neurophysiological monitoring was performed using an electroencephalogram, motor evoked potentials, and somatosensory evoked potentials. A total of 106 laser speckle flowmetry measurements were performed. The mean ± standard deviation blur rate - reflecting relative blood flow - was 32.2 ± 12.7 arbitrary units (AU) before carotid clamping and 19.2 ± 14.3 AU after carotid clamping. After release of the carotid clamp, mean blur rate returned to 31.1 ± 11.8 AU. Similar trends were shown for cerebral oxygen saturation. The correlation coefficient between the reduction ratio of mean blur rate and reduction ratio in cerebral oxygen saturation before and during carotid clamping was 0.69. Further, an increased reduction ratio of mean blur rate was associated with neurophysiological events detected in electroencephalograms, motor evoked potentials, and somatosensory evoked potentials.

Another study by Niemann et al. [5] investigated laser speckle contrast imaging in 9 patients having carotid endarterectomy under local anesthesia. In this study, laser speckle contrast imaging was used to measure blood flow on the patients’ forehead, specifically the ipsilateral and contralateral supraorbital area and the glabella. The effects of common carotid artery clamping and reperfusion of the external and the internal carotid artery on cutaneous blood flow were investigated. As expected, cutaneous blood flow decreased from mean ± standard deviation 334 ± 135 to 221 ± 109 AU (p = 0.023) in the ipsilateral supraorbital region, and from 384 ± 151 to 276 ± 107 AU (p = 0.023) in the glabella during common carotid artery clamping, whereas contralateral supraorbital cutaneous blood flow did not show significant changes. Unexpectedly, after reperfusion of the external carotid artery a trend towards normalization of cutaneous blood flow was observed, while the subsequent reperfusion of the internal carotid artery (anatomically supplying the monitored cutaneous area) was followed by a smaller increase in cutaneous blood flow. Cutaneous blood flow was not observed during isolated external carotid artery clamping. The authors were surprised by the increase in cutaneous blood flow after reperfusion of only the external carotid artery, and concluded that it was not possible to distinguish specific areas perfused by the external and internal carotid artery.

Together, these two studies show that laser speckle contrast imaging is feasible—and may provide important information on regional blood flow during carotid endarterectomy. Whether this information is clinically important—especially when regional oxygen saturation is already monitored with NIRS—will need further investigation. Therefore, future research is necessary to establish the role of laser speckle contrast imaging in guiding diagnostics or even treatment.

Multispectral imaging uses the visible bands of the electromagnetic spectrum to obtain a reflectance pattern and therefore not only allows measurement of blood flow, but also non-invasive non-contact measurement of regional oxygen saturation. In a prospective observational study with 58 healthy volunteers, Bruins et al. [6] investigated whether multispectral imaging detects changes in regional oxygen saturation during a vascular occlusion test (VOT). NIRS was used as a reference measurement for regional oxygen saturation. For the VOT an upper-arm cuff was inflated until regional oxygen saturation determined with NIRS decreased to 40%. At the same time multispectral imaging was performed. The oxygen saturation measurements obtained with multispectral imaging showed a typical desaturation and recovery slope as well as a post-occlusive hyperemia - just like the regional oxygen saturation curve of NIRS. Even though multispectral imaging measurements showed the expected trends, there was only a weak to moderate correlation between NIRS and multispectral imaging regional oxygen saturation measurements. The authors speculate that the observed differences may be in part explained by the different measurement depths—deeper tissue for NIRS and superficial tissues for multispectral imaging—and their different microvascular structure. Multispectral imaging is a relatively novel method for non-invasive and non-contact regional oxygen saturation measurements. The concept is promising as multispectral imaging, in contrast to NIRS, allows measuring regional oxygen saturation in multiple or selected regions of interest. Future research will have to show if this new method can be implemented into clinical practice.

Technical and methodological limitations have yet prevented the use of handheld vital microscopy in clinical practice. Handheld vital microscopy allows direct real-time visualization of the sublingual capillary network—possibly at the bedside. Until now, major limitations of this method were the difficult image acquisition and their complex off-line analysis. In a method comparison study, Coppel et al. [7] compared image quality of the analogue MicroScan camera and its successor, the MicroScan USB3 camera (both Microvision Medical; Amsterdam, The Netherlands). A total of 60 10-s videosequences (30 videosequences with each device) were obtained in healthy volunteers. All videos were obtained by two investigators according to current guidelines for obtaining videosequences using handheld vital microscopy [8], and were rated according to the Microcirculation Image Quality Score based on illumination, duration, focus, content, stability, and pressure [9]. The novel MicroScan USB3 camera had superior rating for illumination, focus, and pressure indicating overall better image quality. These results may be explained by technical advancements, e.g. better camera resolution, adjustable frame, and reduced weight, which facilitates stable measurements. Even though the number of analyzed video sequences was limited, this study indicates technical advancements in the field of handheld vital microscopy. With recent validation of an automated analysis software [10], it will be interesting to see whether handheld vital microscopy can take the next steps to clinical real-time application at the bedside.

In a prospective observational study in 79 septic shock patients, Xu et al. [11] investigated the relationship between transcutaneous oxygen tension measured with a Clark pO_2_ electrode and blood lactate clearance. The measurements were performed within the first hour of treatment in the intensive care unit and again after 6 h. Additionally, an oxygen challenge test during which inspiratory oxygen fraction was increased to 1.0 for 10 min was performed. Patients were divided into a low lactate clearance group (< 20% over the 6 h study period, n = 46) and a high lactate clearance group (≥ 20%, n = 33). Differences in transcutaneous oxygen tension (between baseline and at 6 h) (r = 0.59), absolute transcutaneous oxygen tension at 6 h (r = 0.48), and lowest transcutaneous oxygen tension (r = 0.32) correlated moderately with lactate clearance. There were no important differences in transcutaneous oxygen tension between patients with high and low lactate clearance at baseline. At 6 h, mean ± standard deviation transcutaneous oxygen tension was 62 ± 22 mmHg in the high lactate clearance group and 44 ± 16 mmHg in the low lactate clearance group, indicating a clinically important difference. The oxygen challenge test resulted in higher oxygen tension values in the low lactate clearance group compared to the high lactate clearance group. Even though this study showed a correlation between transcutaneous oxygen tension and lactate clearance in septic shock patients, it remains unknown how this may affect clinical practice—as lactate clearance can be easily measured with blood gas analysis. It has been well investigated that a low lactate clearance is associated with poor outcome [12]. The clinical importance of monitoring transcutaneous oxygen tension will need further investigation.

## Plethysmography: more than SpO_2_ monitoring alone?

Plethysmography is a simple and low-cost optical technique that can be used to detect blood volume changes in the microvasculature [13]. The technology has been used for decades in a wide range of commercially available medical devices for measuring oxygen saturation (pulse oximetry). However, plethysmography may reveal more than just the peripheral arterial oxygen saturation (SpO_2_).

### SpHb monitoring

Using a proprietary algorithm that applies multi-wavelength analysis (Rainbow, Masimo Corp., Irvine, USA), multiple hemoglobin fractions may be assessed so that total hemoglobin concentration (SpHb) can be monitored non-invasively [14]. Ever since its introduction more than a decade ago, the reliability remains a hot topic, not only in terms of accuracy (bias) and precision (i.e. repeatability) but also in terms of trending ability [15]. Two articles recently published in the JCMC further explored this issue. In a study in 48 patients undergoing open abdominal aortic surgery, De Rosa et al. [16] compared SpHb readings with laboratory hemoglobin (Hb) values. Interestingly, the authors investigated point-of-care blood gas analysis derived Hb values as well—which is often considered as the clinical standard. The authors found a mean bias for SpHb of 1.63 (± 0.05) g dL^−1^—with 95% limits of agreement of 0.85 and 2.40 g dL^−1^. For satellite-lab blood gas analysis, bias was 0.69 (± 0.04) g dL^−1^, with limits of agreement between 0.07 and 1.30 g dL^−1^. The authors conclude that SpHb has a limited reliability with respect to absolute values of Hb. Instead, the direction of changes in SpHb accurately followed changes in Hb itself, so that the “trending ability” was considered sufficient by the authors.

Applegate et al. [17] acknowledged the limited reliability of absolute SpHb values, and primarily investigated SpHb trending ability. In 135 patients undergoing surgery, SpHb was compared again with laboratory derived Hb values, as well as with multiple point-of-care (but invasive) alternative monitoring methods. The “direction of change” of SpHb and reference Hb agreed in > 94% of cases. A more in-depth analysis using four quadrant plots revealed that for reference Hb values < 9 g dL^−1^, SpHb indicated well the direction of changes. The authors concluded that when SpHb decreases more than 0.5 g dL^−1^, the decision may be prompted to obtain an invasive reference Hb value.

The results of these studies underline that although SpHb monitoring is non-invasive and continuous, it may in its current version best be used as a trend monitor and trigger for obtaining invasively drawn Hb samples, when changes in SpHb occur.

Finally, Cros et al. [18] took a fairly different approach and studied the effect of implementing SpHb monitoring in the operating room, recovery room, and intensive care unit (ICU) - and demonstrated that the implementation of continuous and non-invasive Hb monitoring based on advanced pulse oximetry may beneficially influence patient outcome. In a “before-after” study-design, the authors investigated whether the use of SpHb together with the use of pleth variability index (PVI, a non-invasive photoplethysmography-derived variable that can be used to assess fluid responsiveness [19]) would reduce blood transfusion requirements and mortality. Two 11-months periods were studied (with n = 9285 patients (before) vs. n = 9431 (after)). In the after-group, clinicians were free to choose whether they would use SpHb and PVI monitoring (n = 3575). Interestingly, the authors observed that in these patients, blood transfusion was performed more frequently in an earlier phase, but with a reduced total amount of transfusions. Also, subtle differences were observed with respect to short-term mortality, indicating a small benefit for those patients monitored with SpHb and PVI. It should be stressed that a few remarks can be made, namely the lack of external-validity (as it is a single-center study) and the possibility of selection-bias (clinicians were free to choose whether to adopt monitoring or not in the “after-group”). Also, since two variables were introduced simultaneously, it remains difficult to discriminate the individual effects of either variable on the chosen outcomes—future prospective studies may further elucidate these issues.

### Oxygen reserve monitoring

The importance of SpO_2_ monitoring is unequivocal and well-established within major guidelines on the basics of perioperative monitoring [20]. SpO_2_ monitoring alone may however not reveal two important instances: *impending* hypoxemia and *inadvertent* hyperoxemia. Here, the oxygen reserve index (ORi), a dimensionless index derived from multi-wavelength pulse co-oximetry that reflects oxygenation in the moderate hyperoxic range [21], may play a role. Two articles recently published in this journal investigated these two aspects.

For *impending hypoxemia*, Tsymbal et al. [22] studied 72 adult patients during induction of general anesthesia - of whom 36 were obese and 36 were non-obese. After pre-oxygenation and induction of general anesthesia, patients were kept apneic until SpO_2_ dropped to 94%. For the purpose of the study, the authors calculated both the ‘ORi warning time’ and ‘SpO_2_ warning time’. The first is defined as the time between an alarm from the ORi monitor and SpO_2_ to drop to 94%, the latter as the time of SpO_2_ to drop from 97 to 94%. The *added* warning time is the time difference between the ORi and SpO_2_ warning time. Interestingly, the added warning time was positive in all cases—meaning that ORi warned earlier than SpO_2_ for impending hypoxemia, and was 46 (95% confidence interval: 36–59) seconds for obese and 87 (95% confidence interval: 77–109) seconds for non-obese patients. The authors conclude that this added time could be clinically relevant and might allow earlier calls for help and assistance, should hypoxemia be impending. The authors performed a comparable study previously in pediatric anesthesia - with similar findings [23]. The algorithm that initiates an ORi alarm remains proprietary and functions as a ‘black box’ for the clinician. Whilst severe desaturation is usually defined as SpO_2_ below 85% for longer than 5 min [24] it may be speculated that ORi monitoring ultimately allows reducing the incidence of hypoxemia by buying the clinician extra time to (re)act and optimize oxygenation and ventilation.

Yoshida et al. [25] investigated instances of hyperoxemia in 20 mechanically ventilated patients under general anesthesia. The authors recorded the inspiratory O_2_ fraction (FiO_2_), ORi, and blood gas analysis-derived PaO_2_ during administration of 0.33 FiO_2_, and during 3 pre-set ORi targets of 0.5, 0.2 and 0.0. The main goal of the study was to assess the relationship between ORi and PaO_2_, and to investigate whether ORi could be used as a measure of hyperoxemia. For PaO_2_ values < 240 mmHg, a reasonable correlation was found (r^2^ = 0.706) - reflecting that absolute ORi values cannot be directly related to absolute PaO_2_ values. Instead, to predict hyperoxemia - defined as PaO_2_ > 150 mmHg - a sensitivity of 95% and specificity of 76% was found, with an optimal ORi cut-off value of 0.21 indicating that ‘excessive’ hyperoxemia may be prevented by maintaining ORi below 0.21. Of note, in analogy with SpHb monitoring, trend analysis of changes in PaO_2_ and consecutive changes in ORi were demonstrated to agree perfectly - indicating that ORi monitoring might be used to reveal changes in oxygenation status too.

### Perfusion index

Finally, pulse co-oximetry continuously provides a measure of peripheral perfusion, called perfusion index (PI). The variation of the PI can be quantified as the PVI (see above). The PI basically reflects the ratio of pulsatile and non-pulsatile light absorbance of the red and infrared light passing through the tissue [19]. This means that if PI is low (e.g. < 1.0), there is a relatively “large” amount of non-pulsatile blood, and vice versa, hence, giving an impression of peripheral perfusion. Although the variable has been available for many years, renewed interest resulted in an increase in publications in which PI was used for the assessment of hemodynamics (e.g. PVI for assessing fluid responsiveness), nociception, and success of peripheral nerve blocks [26]. For the latter, Abdelhamid et al. [27] measured PI at the little finger during and after supraclavicular plexus block. Interestingly, in 9 out of 49 patients in whom ‘ulnar sparing’ occurred - i.e. absence of any sensation in the upper limb apart from ulnar dermatomes - PI did not increase in the little finger. In contrast, PI increased in those patients in whom the block was successful, presumably reflecting the induced altered sympathetic/parasympathetic balance, resulting in vasodilation. This study may pose a readily available clinical use for PI, although it must be stressed that PI is a variable resulting from a complex signal and undergoes (extensive) processing by proprietary algorithms [26]. Since PI values coming from different devices have not been compared as of yet, general cut-off values for example for the determination of “block success” cannot be easily determined.

A promising new tool was investigated in two recent studies in this journal [28, 29]. Here, the authors introduced a device that provides urethral perfusion index (uPI) by plethysmography-equipped urinary catheter. This “simple” and easy-to-use continuous monitoring device may provide important information on abdominal perfusion. Since macro- and microhemodynamics may uncouple to a certain extent [30], monitoring of urethral mucosal perfusion may allow a better understanding of the coupling of macro- and microhemodynamic variables. In the future, this important information on microcirculatory perfusion—will potentially allow the tailoring of hemodynamic management.

In an experimental study investigating the uPI, Cardinali et al. [29] exposed twelve piglets to a series of interventions that mainly led to arterial hypotension (sevoflurane overdose) or hypertension (norepinephrine infusion). Simultaneous measurements of mean arterial pressure (MAP) and uPI were obtained. The most important findings of this animal study were that it appeared that (a) uPI was monitored continuously and reliably and (b) that there was an individual threshold at which the association between MAP and uPI reversed: a positive model coefficient below this threshold, and a negative model coefficient above this threshold - suggesting that the device at least allows the detection of differences in urethral mucosa perfusion induced either by hypotension or hypertension.

In a first-in-human study, Dépret et al. [28] investigated the feasibility and safety of continuous uPI monitoring in critically ill and high-risk surgical patients (n = 28). It was shown that pain scores at insertion and withdrawal of the device, as well as rate of complications, were comparable with those reported for routine urinary catheters. As a secondary outcome, the authors assessed the association between uPI and hemodynamic variables: interestingly, for half of the monitoring time, a positive correlation between MAP and uPI was found, whereas a negative association was observed during other monitoring periods. Future studies should assess the potential use of uPI monitoring for monitoring micro- and macro-hemodynamics, and eventually whether hemodynamic management based on uPI influences patient outcome.

## Tissue oxygenation monitoring in critical illness and non-cardiac surgery

We have already discussed a number of novel devices and clinical variables that are making their way into the expanding field of microcirculation monitoring. NIRS already has an established role in this field, and it is worth mentioning the most recent findings reported in critically ill and non-cardiac surgery patients. Filho et al. [31] performed a pilot study in ICU patients to compare different methods for the assessment of peripheral microcirculatory dysfunction using NIRS. In the first 24 h after ICU admission, NIRS-derived oxygenation values - both static and dynamic (after a vascular occlusion test) - recorded at the thenar eminence were lower in patients with circulatory shock as compared to patients without shock. Interestingly, other validated indices of peripheral tissue perfusion (CRT, PI, lactate, and skin temperature gradient) could not discriminate patients with shock from those without shock. Hence, peripheral NIRS monitoring might be an adjunctive tool in the evaluation of tissue perfusion in critically ill patients, allowing the early detection of microcirculatory dysfunction.

Maintaining adequate tissue oxygen delivery is not only important in ICU patients, but it is also a priority in anesthetic management of surgical patients. As modern surgery moves towards minimally invasive approaches, the anesthesiologist faces new challenges in maintaining adequate tissue perfusion: for example, an increased intra-abdominal pressure due to pneumoperitoneum in laparoscopy, or an increased cerebral blood flow during surgery in the extreme Trendelenburg position, have the potential to compromise tissue oxygen delivery and organ perfusion.

Kamata et al. [32] hypothesized that pneumoperitoneum would cause a decrease in cerebral and renal tissue oxygenation in infants undergoing laparoscopic pyloromyotomy. They showed that cerebral oxygenation decreased, and cerebral fractional oxygen extraction increased from surgical incision to the end of laparoscopy, even though the values recorded at the end of laparoscopy were comparable to the baseline values recorded before anesthesia induction. Both renal tissue oxygenation and renal fractional tissue oxygen extraction did not change during pneumoperitoneum, supporting the safety of laparoscopy in this clinical context.

In another study, Zdravkovic et al. [33] monitored NIRS-derived oxygenation values (and their response to ischemic provocation by a vascular occlusion test) at the forearm and at the calf, aiming to explore the effects of combined spinal-general anesthesia and gynecological laparoscopy. Performing either high- or low-dose spinal analgesia (levobupivacaine plus sufentanil) led to a decrease in the post-ischemic recovery NIRS values measured at the calf before general anesthesia induction. Interestingly, during laparoscopy, the post-ischemic recovery was further reduced only in patients with high-dose spinal analgesia, likely due to the sympatholytic effect of high-dose spinal analgesia leading to an increased number of perfused capillaries. The intriguing interpretation by the authors was that the slower post-ischemic recovery should not be considered as reflecting an impaired microcirculatory function, but instead a more hemodynamically favorable state related to an increased capillary bed, raising the hypothesis that spinal analgesia might have a role in improving microcirculatory function under certain surgical and anesthesiologic conditions. Additionally, no differences between study groups were found in NIRS-related values measured at the forearm, i.e. a region where the vascular bed was not affected by the spinal analgesia.

It is worth noting that in both the two aforementioned studies, two sites of NIRS monitoring were investigated (cerebral and renal [32] versus calf and forearm [33]), and different findings were reported for each site. This implies that the monitoring sites are often not interchangeable, as other authors hypothesized and demonstrated: Fan et al. [34] found a large discrepancy and an inconsistent correlation between cerebral and somatic tissue oxygenation in 205 surgical patients having robotic hysterectomy or spine surgery. These findings support the conclusion that NIRS is not a “one size fits all” monitoring, and NIRS-derived values should be carefully interpreted according to the underlying physiology of the monitored tissue.

Beck et al. [35] hypothesized that an impairment in cerebrovascular autoregulation due to elevated intracranial pressure could be found in patients undergoing robot-assisted radical prostatectomy in the extreme head-down (Trendelenburg) position. Cerebral autoregulation was continuously assessed by calculating the cerebral oxygenation index, but no differences in cerebral autoregulation were demonstrated between robot-assisted (head-down position) and open radical prostatectomy (supine position). A transient increase in the cerebral oxygenation index was observed during the intraoperative period in both groups, indicating that factors other than positioning could contribute to a transient impairment of cerebral autoregulation. Additionally, increasing age and a larger difference between baseline and intraoperative MAP were associated with impaired autoregulation, regardless of the surgical technique.

During major vascular surgery, clinicians are well aware that maintaining adequate perfusion of the spinal cord is crucial, since spinal ischemia might have disastrous consequences. In an interesting report of two cases of thoraco-abdominal aortic repair, Giustiniano et al. [36] used NIRS to monitor regional tissue oxygenation of the paraspinous muscles as an index of spinal cord perfusion. While in one patient intraoperative NIRS values remained within ± 20% compared to baseline values, and no spinal cord injury occurred, in another patient NIRS values persistently decreased > 20% below baseline values, and spinal cord injury was immediately manifest at the postoperative neurologic evaluation as a palsy of the lower limbs. However, as indirectly suggested in another study, it is not certain if paraspinal oxygenation accurately tracks spinal cord oxygenation. In fact, Vanpeteghem et al. [37] found that phenylephrine administration increased paraspinal oxygenation, while ephedrine decreased it. This was in contrast to the effect of these drugs on cerebral oxygenation, which decreased with phenylephrine and remained unchanged with ephedrine administration, in agreement with previous studies [38, 39]). One explanation suggested by the authors is that vasoconstriction of the spinal vessels results in a redistribution of blood flow to the spinal muscles, and paraspinal oxygenation therefore would not represent spinal cord oxygenation. Another possible explanation is the presence of different subtypes of adrenergic receptors in the spinal and cerebral vasculature. In both cases, exploration of the mechanisms behind this phenomenon warrants future research.

## Tissue oxygenation monitoring in cardiac surgery

The main topics addressed in the past two years were:

- The accuracy of the NIRS measurements in patients with different skin color.
- The usefulness of tissue oxygenation monitoring (measured over the forehead and thenar eminence) for predicting the occurrence of postoperative neurocognitive disorders (e.g., delirium and cognitive decline) and in measuring microvascular reactivity.

### Tissue oxygenation measurements and ethnicity

In the article published by Stannard et al. [40] in the April 2021 issue of the JCMC, the authors investigated if the patient’s skin pigmentation affects the cerebral regional oxygenation (rSO_2_) measurements. They conducted a multiple logistical regression analysis using retrospectively collected rSO_2_ data and self-reported ethnicity data collected from 4267 patients undergoing on-pump cardiac surgery. After adjusting for the perioperative variables, they found less than 2% differences in rSO_2_ readings between distinctive racial groups. This finding contrasts with the article published by Sun et al. [41], who demonstrated an 8% lower baseline in patients with African heritage (i.e. darker skin). This discrepancy can be partially explained by the difference in monitor (and technology) used in the two studies. The authors also found that a mean pre-bypass rSO_2_ < 63% was an independent factor associated with increased 30-day mortality.

### Tissue oxygenation and neuropsychological functioning

Genbrugge et al. [42] published a study in the February 2021 edition of JCMC investigating the influence of electrical cardioversion (ECV) in 60 patients with atrial fibrillation (AF) on cerebral rSO_2_ and long-term neuropsychological functioning. This was in response to a publication describing that AF is an independent risk factor for cognitive impairment even in the absence of manifest stroke [43]. The authors hypothesized that returning to a sinus rhythm would improve the short-term rSO_2_ and wanted to determine if this rSO_2_ increase is persistent and limits cognitive decline after AF. In 50 patients with successful ECV and ten patients with an unsuccessful ECV, neuropsychological tests and tissue oxygenation measurements were conducted before, during, and 4 to 6 weeks after ECV. The authors found that the successful ECV led to an immediate increase in rSO_2_, which was also positively correlated with a concomitant increase in blood pressure. Further, even though the restoration of sinus rhythm improved the patients’ reported quality of life, the rSO_2_ increase did not persist at 4 weeks after the event, and it did not influence neuropsychological functioning at follow up.

In the August 2020 issue of the JCMC, Soh et al. [44] investigated the predictive value of rSO_2_ and the mean velocity flow (MVF) in the middle cerebral artery, measured using transcranial doppler, on the occurrence of postoperative delirium. They hypothesized that the baseline, preoperative assessment of these cerebral hemodynamic variables might provide an estimation of “patients individual reserve against cerebral hypoperfusion” and thereby might identify patients at high risk of developing postoperative delirium. In this prospective observational trial including 113 patients undergoing valvular heart surgery, baseline cerebral hemodynamics were noted before the induction of anesthesia, and the occurrence of postoperative delirium was assessed daily with the confusion assessment method for the ICU during the first 7 postoperative days.

After multivariate logistic regression analysis, only low preoperative rSO_2_ (< 60%) remained an independent predictor of delirium, while contrary to the author's expectation, the middle cerebral artery MVF did not. They attributed their finding to the fact that MVF only describes one side of the oxygen supply/demand equation by enabling a rough assessment of oxygen delivery without measuring the cerebral metabolic demands.

Previously, Cho and colleagues [45] had demonstrated that the microvascular circulation, estimated using thenar rSO_2_ measurement during a VOT, is less affected by general anesthesia with desflurane–remifentanil compared to propofol–remifentanil anesthesia in patients undergoing thoracoscopic surgery [6]. In an article published in the October 2021 edition of JCMC [46], they conducted a similar study comparing the effects of desflurane vs. sevoflurane-based anesthesia on microvascular reactivity in patients undergoing off-pump coronary artery bypass graft surgery. Thenar rSO_2_ was monitored and VOTs were performed before induction of anesthesia, before and after a coronary artery anastomosis, and at the end of surgery. Thenar rSO_2_ and the recovery slope after VOT were similarly decreased at the end of surgery compared to the baseline measurement with both anesthetics. The macro-hemodynamic variables, biochemical measures, and in-hospital adverse events were similar in both groups. The authors conclude that both anesthetics similarly influenced microvascular hemodynamics.

In the April 2020 issue of the JCMC, Holmgaard et al. [47] published a retrospective sub-analysis of data collected for the FLuid responsiveness prediction using EXtrasystoles (FLEX) trial [48]. They wanted to compare the influence of a standardized fluid bolus (5 mL kg^−1^) on cardiac output, stroke volume (SV), and cerebral oxygen saturation (ScO_2_) in patients who responded with an SV increase (> 10%) vs the non-responders. They found that increasing cardiac output with a fluid bolus by itself did not improve ScO_2_ (neither in fluid responders nor in non-responders), even though a weak correlation was found between relative changes in cardiac output and ScO_2_. Nevertheless, fluid bolus remains an essential method of increasing cardiac output to maintain adequate cerebral perfusion and hence ScO_2_ in fluid responsive (or volume deficient) patients.

## Conclusion

This review summarizes new evidence on tissue oxygenation and microcirculation as published in the last 2 years in the JCMC. While some techniques are already currently used as routine monitoring (e.g. cerebral oxygenation using NIRS), other techniques still have to find their way into clinical practice. Therefore, further research is needed, particularly regarding outcome measures and cost-effectiveness, since introducing new technology is always expensive and should be balanced by downstream savings. The JCMC is glad to provide a platform for such research.

## Data Availability

Available at a reasonable request.
